# Whole-Genome Sequencing of a *Campylobacter jejuni* Strain Isolated from Retail Chicken Meat Reveals the Presence of a Megaplasmid with Mu-Like Prophage and Multidrug Resistance Genes

**DOI:** 10.1128/genomeA.00460-16

**Published:** 2016-05-26

**Authors:** Daya Marasini, Mohamed K. Fakhr

**Affiliations:** Department of Biological Science, The University of Tulsa, Tulsa, Oklahoma, USA

## Abstract

Genome sequencing of *Campylobacter jejuni* strain T1-21 isolated from retail chicken meat revealed the presence of a chromosome of 1,565,978 bp and a megaplasmid of 82,732 bp that contains Mu-like prophage and multidrug resistance genes. This is the first reported sequence of a *Campylobacter* megaplasmid >55 kb.

## GENOME ANNOUNCEMENT

*Campylobacter jejuni* and *Campylobacter coli* are the two major species associated with human infections (1). In the last few years, our laboratory was successful in isolating a large number of *C. jejuni* and *C. coli* strains from retail chicken livers and gizzards (2), beef livers (3) and poultry meat (4). A large number of these strains were shown to harbor large plasmids (5). Recently, the complete genome sequence of a *C. jejuni* isolate from beef liver showed the presence of two plasmids (44 kb and 4.4 kb) with a chromosome of 1.7 Mb in size, in which the larger plasmid (44 kb) contained the *C. jejuni* integrated elements (6). We report here the whole-genome sequence of *C. jejuni* T1-21, which was isolated from retail chicken meat and shown by agarose gel electrophoresis to contain a large plasmid of approximately 80 kb in size.

Genomic DNA was isolated from a microaerophilic 72-h culture grown in Muller-Hinton broth with 5% laked horse blood using the DNeasy blood and tissue kit (Qiagen, Valencia, CA). Next-generation sequencing was performed in a MiSeq platform using the Illumina V2 reagent kit with 2 × 150 cycles (Illumina, Inc., San Diego, CA), with a coverage of 121×. A library was constructed using the Nextera XT sample preparation kit (Illumina, Inc.). The sequence analysis and assembly were carried out using CLC Genomics Workbench 7.5.1 and the Microbial Genome Finishing Module version 1.4 (Qiagen, Inc.).

The genome of *C. jejuni* T1-21 contained a circular chromosome of 1,565,978 bp in size, along with a megaplasmid of 82,732 bp. The chromosome is slightly smaller in size than the closest reference chromosome of strain *C. jejuni* F38011, which is 1,691,939 bp long. The complete genome contained 1,754 genes, of which 1,645 coding sequences (CDSs), 60 pseudogenes, and 49 genes coding for RNAs were present.

Most of the previously sequenced *Campylobacter* plasmids were pTet plasmids ranging in size from 40 kb to 55 kb and have tetracycline resistance genes. The 82,732-bp megaplasmid sequenced in this study contains a total of 105 complete genes and a pseudogene, as annotated by the NCBI Prokaryotic Genome Annotation Pipeline. To our knowledge, this is the first report of a complete sequence of a megaplasmid >55 kb reported in *Campylobacter*. The multidrug resistance portion of the plasmid was shown to carry streptothricin, tetracycline, aminoglycoside, and hygromycin resistance genes and was similar to the previously sequenced aminoglycoside-resistant plasmid pN29710-1 of 55 kb in size (7). Our megaplasmid also contains genes encoding bacteriocin, virulence-associated protein, and *cag* pathogenicity island protein. It also contains a 45-kb portion that contains Mu-like prophage genes. Although the presence of a *C. jejuni* integrated element (CJIE) was previously reported in the chromosomes of *C. jejuni* and *C. coli* (8), this is the first reported *Campylobacter* plasmid of this large size having CJIE with Mu-like prophage.

### Nucleotide sequence accession numbers.

The GenBank accession numbers of the chromosome and the plasmid of the *C. jejuni* T1-21 strain are CP013116 and CP013117, respectively.
